# The relationship between perceived stress and prolonged grief disorder among Chinese Shidu parents: effects of anxiety and social support

**DOI:** 10.1186/s12888-023-05206-9

**Published:** 2023-10-03

**Authors:** Jiexi Xiong, Hongfei Ma, Ruiyao Ma, Tianhui Xu, Yang Wang

**Affiliations:** Department of Social Medicine, College of Health Management, China Medical University, No. 77 Puhe Road, Shenyang North New District, Shenyang, Liaoning 110122 P.R. China

**Keywords:** Shidu parents, Perceived stress, Anxiety, Social support, PGD, Moderated mediation model

## Abstract

**Background:**

Shidu parents refer to the couple who have lost their only child and have not given birth or adopted another child in China. The number of Shidu parents is increasing annually. The aim of this research was to examine the mediating role of anxiety and the moderating role of social support between perceived stress and prolonged grief disorder (PGD) among Chinese Shidu parents.

**Methods:**

A cross-sectional study was carried out with 505 participants who completed a questionnaire including the Prolonged Grief Questionnair-3 (PG-13), the Perceived Stress Scale-10 (PSS-10), the Self-Rating Anxiety Scale (SAS) and the Duke-UNC Functional Social Support Questionnaire (FSSQ). SPSS PROCESS macro was employed to examine the mediating role of anxiety and the moderating role of social support.

**Results:**

The mediation analysis showed anxiety partially mediated the link between perceived stress and PGD, and the proportion of mediation of anxiety was 39.22%. The moderated mediation analysis revealed the second stage of mediating effects of anxiety on the link between perceived stress and PGD was moderated by social support. Specifically, compared with Shidu parents with higher social support, the association between anxiety and PGD was closer for those with lower social support.

**Conclusions:**

The moderated mediation model can broaden our understanding of how and when perceived stress, anxiety and social support work together to affect PGD. The interventions aimed at improving mental health of Chinese Shidu parents need to work on reducing stress and enhancing social support.

## Background

One-child policy was carried out by the Chinese government between 1979 and 2015, which aimed to slow population growth and promote economic development. The policy effectively brought down China’s total fertility rate but increased the risk of one-child households becoming childless [1]. Shidu parents are characterized as the couple who have lost their only child and have not given birth or adopted another child [2]. According to data from 2013 national census in China, there were at least one million Shidu families, and the number was growing by 76,000 families annually [3]. After losing the only child, Shidu parents have to deal with stress from traumatic loss and Chinese unique societal and cultural context, which can cause physical and mental illnesses [4]. On the one hand, Chinese culture is family-oriented [2]. The families are so extremely child oriented that children are regarded as the source of well-being and affectional bonds for the whole family [5]. Losing the only child indicates the parents have no offspring, which puts them into a very distressing situation, as a Chinese saying, “white hair sends black hair”. On the other hand, Confucianism believes that it is extremely unfilial to have no offspring, and individuals without children are culturally stigmatized as “juehu”, meaning those who are going extinct [6]. It makes the death of children becoming an unmentionable topic associated with many Chinese superstitions and customs [4]. Finally, generally speaking, children take the responsibility to care for their elderly parents, which is not only the requirement of Chinese traditional filial piety, but also an obligation enforced by law in China. Yet the death of the only child will invalidate all the parents' financial and emotional investment on their children, and ultimately increase their economic and social burdens in old age [5]. To our knowledge, there are numerous bereavement studies on the loss of relatives and spouses, but very few studies are specifically about the loss of an only child, especially in China where the culture is completely different from that of western countries.

PGD is a disorder in which the bereaved experience a continuous and pervasive grief response after the death of a close person, and it is characterized by yearning for the dead or enduring preoccupation with the dead along with intense emotional pain for over 6 months [7]. The prominent emotional characteristics of PGD are intense longing for the deceased and avoidance of thinking about the loss, and other common symptoms of PGD include intrusive thoughts about lost situations, guilty, avoidance of past shared activities and inadequate adjustment to life after the loss [8]. Additionally, as a qualitatively unique grief-related disorder, PGD is distinct from depression, anxiety and PTSD [9–11]. To be specific, yearning and emotional pain load highly on the PGD factor but not on depression, anxiety or PTSD factors, whereas sadness loads highly only on a depression factor, and feeling nervous and fearful as well as heart pounding load highly only on an anxiety factor [10, 11]. As has been documented by some studies that PGD was related to increased rates of panic disorder, alcohol abuse, lifetime suicide attempts, functional impairment, poor sleep quality, and reduced the quality of life [12–14]. The prevalence of PGD has been estimated to be 1.8%–13.9% among general bereaved people, and the range of the prevalence among bereaved parents was 10% to 30% in various counties [9, 15–24]. Therefore, there is an urgent need to further explore the mechanisms that may affect PGD and to develop measures to prevent or alleviate PGD symptoms among Chinese Shidu parents. However, due to the difficulties in surveying and obtaining data on the Shidu population, there are few studies researching on PGD in them.

The death of a close person is one of the most stressful experiences in one’s life, which can seriously affect physical and mental health [25]. Perceived stress reflects an individual’s global subjective evaluation to the stress level which caused by objective events [26]. In addition to the stressor of traumatic loss, Shidu parents will also cope with stress from the cultural stigmatization, social isolation and financial adversity in daily life after losing a child due to Chinese unique social and cultural surroundings [4]. According to the stress and coping theory introduced by Lazarus and Folkman, when a major loss occurs, both the loss itself and the daily troubles generated in living with the loss can be appraised as stressful, and then the stress can be managed through coping processes in an attempt [27, 28]. Some coping methods are problem-focused, aimed at managing or altering problems, while others are emotion-focused coping and aim to manage emotional responses to problems, with the grief response being one emotion-focused coping process that attempts to manage stress generated by the bereavement [27, 28]. A previous literature indicated that level of grief among individuals who were caregivers for a loved one with dementia increased with the severity of the care recipient's dementia, and higher levels of grief were significantly associated with stress [29]. Ward et al. [30] reported that bereaved participants with PGD had higher levels of stress than other bereaved participants without PGD. Although the relation between stress and grief is mentioned, the relation between perceived stress and PGD as well as the mechanism underlying this relationship among Shidu parents is still unclear. Therefore, the investigation aimed to study mediating mechanisms and moderated mediation effects in the relation between perceived stress and PGD.

### The mediating role of anxiety between perceived stress and PGD

As a negative emotion, anxiety is triggered by perceived threats that may come from the environment (e. g. social occasions or new locations) or from within oneself (e. g. unusual physical sensations) [31]. Although perceived stress and anxiety are both emotional responses with many similarities between symptoms, perceived stress is caused by an existing external stress-causing factor or “stressor”, when the stressor disappears, so does perceived stress; whereas individuals with anxiety continue to feel pressure even after the stressor is gone. According to the biobehavioral attachment-based model of prolonged grief proposed by Shear and Shair [32], the attachment system established in infancy is activated under stress for regulating emotions to cope with stress and establish personal security. However, after the death of the attachment figure, great bereavement-related stress activates the attachment system, and individuals with insecure attachment style may experience anxiety symptoms due to their inability to update old attachment schemas and form new attachment relationships in time [32]. The anxiety symptoms are focused on the unease related to the loss of the important refuge and secure base provided by the attachment figure, as well as the confrontation with death. This painful emotion further activates the attachment system and inhibits engagement in social life, and then the distress escalates, interfering with and complicating the grieving process [32]. Some previous studies in other populations have explored the relationship between perceived stress and anxiety. The study among Chinese college students supported that perceived stress could significantly positively predict anxiety [33]. Li et al. [34] also reported higher levels of perceived stress was strongly connected with increased risk of anxiety in breast cancer patients. Regarding to anxiety and PGD, previous studies have reported anxiety was significantly correlated with PGD [18, 35]. The study in bereaved caregivers by Aoun and colleagues [16] displayed when participants had anxiety, the risk of PGD was 8 times higher than those without anxiety. Thus, we propose a hypothesis that anxiety mediate the relation between perceived stress and PGD.

### The moderating role of social support

Social support refers to emotional, informational and instrumental assistance provided to the individual by primary and secondary group members [36]. The buffering model indicates that when people face stress, social support plays a buffering role through people’s cognition to reduce the negative effects of stress on them and protect their physical and mental health [37]. Cao et al. found that both perceived social support and objective social support could reduce anxiety among Chinese Shidu parents [5]. Results of a study by Razurel et al. [38] showed during the prenatal period, parturient received esteem and information support from their mother as well as information support from their friends, which could moderate the association between stress and prenatal anxiety. Zou et al. [39] found the relation between perceived stress and anxiety was moderated by social support in Chinese frontline COVID-19 medical staff, specifically, among individuals with low social support, higher perceived stress was related to more anxiety. Furthermore, researchers have reported perceived social support moderated the link between attachment anxiety and health outcomes, and less anxious individuals’ health benefited from high support [40]. An empirical study also revealed social support could moderate the association between anxiety and depression in survivors of Wenchuan Earthquake [41]. Social support may also help individuals cope with PGD. Song et al. [42] found PGD symptoms of Shidu parents were negatively correlated with social support. The study in caregivers of people with motor neurone disease revealed social support was a critical predictive factor of PGD, and increasing social support could reduce psychological distress [16]. These findings coincide with the biobehavioral attachment-based model of prolonged grief emphasizing that social support from others and the social environment can compensate for the bereaved's temporary withdrawal from interpersonal interactions [32]. A good social support system can help the bereaved maintain a sense of control, stop avoidance, and gently encourage them to think about future plans and adapt to life without the deceased [32]. Therefore, we hypothesize social support can moderate the direct and indirect pathways of the mediating model.

**Fig. 1:**
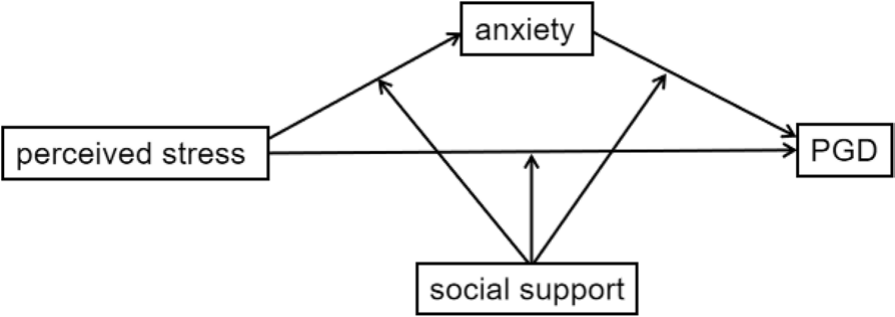
The hypothesized moderated mediation model

To sum up, the current study pursued three goals: 1) to explore whether perceived stress significantly and positively associated with PGD, 2) to determine whether anxiety mediate the relationship between perceived stress and PGD, 3) to examine whether the direct and the indirect effects of perceived stress on PGD are moderated by social support (see Fig. 1). This is the first investigation to examine the mediating role of anxiety and the moderating role of social support in the relation between perceived stress and PGD among Chinese Shidu parents. We hope this study will help the government and primary health care institutions formulate targeted interventions to improve mental health of Chinese Shidu parents in the future.

## Methods

### Participants and procedures

The cross-sectional study was conducted in Shenyang, a city in northeast China, from March 2017 to September 2017. We selected five urban districts in Shenyang and randomly collected 15 communities in each district. All Shidu parents who met the following inclusion criteria in these communities were recruited as participants: 1) lived in the community for over half a year; 2) lost the only child and had no biological or adopted another child at the time of the survey; 3) could communicate in Chinese clearly. The exclusion criteria was having severe mental illness (e.g. mental retardation, Alzheimer’s disease). We used the following formula to calculate the sample size: $n=\frac{{Z}_{1-a/2}^{2}p\left(1-p\right)}{{d}^{2}}$. The prevalence of PGD in bereaved parents ranged from 10% to 30% around the world [20–24], we chose 20% as the expected prevalence. The sampling error allowed in this study was d = 0.05, and 95% CIs was adopted, so $Z_{1-a/2}$ = 1.96. We calculated the minimum sample size: *n* = [1.96^2^ × 20% × (1-20%)]/(0.05)^2^ ≈ 246. Due to the particularity of the loss, we assumed the response rate of the subjects was 70%, which required the sample size: *n* = 246/70% ≈ 352. Community family planning commissioners, who had access to all Shidu families in the community, registered participants who met the inclusion criteria. The commissioners explained the purpose of this survey and invited Shidu parents who were willing to join in the survey to the community office to fill out self-report questionnaires. Participants provided written informed content before the investigation, and interviewers explained the purpose and significance of the investigation. Community family planning commissioners could assist in providing clarification if participants had any questions. When subjects had trouble in completing questionnaires by themselves, face-to-face interviews were used to obtain the answers. Before data collection, in order to make sure a standardized process, all community family planning commissioners were trained for two days. After the investigation, subjects were paid 100RMB as compensation. In total, a sample of 595 subjects was surveyed. Of these, 79 refused to join in. Among the 516 subjects, 11 were not included in the analysis because more than 30% of the data were missing. As a result, 505 parents remained for analysis, and effective response rate was 84.87%. GPower was used to conduct power analysis of the sample size, when α = 0.05, effect size = 0.3, the power of the sample size was 0.99. There were no significant demographic differences between the 505 included participants and the nonparticipants. The missing data were lost completely at random.

The study was approved by the Ethics Committee of China Medical University, and all the research procedures were conducted in accordance with ethical standards.

### Demographic characteristics and bereavement-related information

Demographic characteristics consisted of parents’ gender, age, marital status, education level, employment, chronic disease, annual household income, debt, religious belief, marital satisfaction. Bereavement-related information included gender and age of the deceased child, time since the child’s death, cause of death and whether the participant had a grandchild. If participants had been diagnosed with any of chronic diseases (e. g. hypertension, diabetes, cardiovascular disease, chronic hepatitis, ulcer, gout and arthritis), chronic disease was recorded as ‘yes’. Debt, religious belief and whether the participant had a grandchild were recorded as ‘yes’ or ‘no’. Cause of death was divided as ‘accident’, ‘acute disease’, ‘chronic disease’ and ‘mental disease’.

### Assessment of prolonged grief disorder

PGD symptoms were measured by the Prolonged Grief Questionnair-13 (PG-13), a screening instrument to diagnose PGD according to the criteria defined by Prigerson [10]. It consists of 13 items addressing separation distress (1-2); cognitive, emotional and behavioral symptoms (3-11); duration criteria (12); and impairment criteria (13). Item 1 to 11 are rated on a 5-point Likert scale (1 = never, 5 = several times a day/overwhelmingly), and items 12 and 13 are yes/no questions. The diagnostic criteria for PGD include the following: 1) the individual has lost a significant other; 2) separation distress: at least one item with a rating of 4 or higher; 3) cognitive, emotional and behavioral symptoms: at least five items with a rating of 4 or higher; 4) duration criteria: item 12 must receive ‘yes’ response that means separation distress should last for more than 6 months after the death; 5) impairment criteria: item 13 must be answered as ‘yes’ that means being defective in social, occupational and other crucial aspects [10]. The Chinese version of PG-13 has been proved to have satisfactory psychometric properties [9]. In the investigation, the internal consistency coefficient for the scale was 0.91.

### Assessment of perceived stress

Perceived stress was assessed by the Perceived Stress Scale-10 (PSS-10), which evaluated the degree of stress that a participant experienced over the past month [43]. It consists of four positive items (4, 5, 7 and 8) and six negative items (1, 2, 3, 6, 9 and 10). Each item is rated on a 5-point Likert scale, ranging from 0 (never) to 4 (all of the time). The total scores range from 0 to 40, and higher scores indicate more stress. This scale has shown adequate validity and reliability in Chinese population [44]. Cronbach’s alpha was 0.86 in this study.

### Assessment of anxiety

Anxiety symptoms were assessed by the 20-item Self-Rating Anxiety Scale (SAS) [45]. Items are scored on a 4-point Likert scale ranging from 1 (rarely or none of the time) to 4 (most or all of the time), according to the frequency of anxiety symptoms during the past week. Five items (5, 9, 13, 17 and 20) are reverse scored. The total scores of this scale range from 20 to 80, and scores of 50 or more is considered as the presence of anxiety symptoms. The higher the total scores, the more severe anxiety symptoms. The Chinese version has shown good validity and reliability [46]. Cronbach’s alpha was 0.91 in the current study.

### Assessment of social support

We used the Duke-UNC Functional Social Support Questionnaire (FSSQ) to evaluate social support, which is an 8-item questionnaire [47]. Each item is rated on a 5-point Likert scale, ranging from 1 (much less than I would like) to 5 (as much as I would like). The scale provides total scores between 8 and 40 points, and the higher the total scores, the greater social support. The scale has shown sufficient validity and reliability in Chinese population [48]. The internal consistency of FSSQ in the study was 0.87.

### Statistical analysis

All the analyses were performed with IBM SPSS Statistics Version 23.0. Descriptive data were presented as frequency, percentages, mean scores and standard deviations. Independent t-test or one-way ANOVA was performed to compare the PGD scores across demographic characteristics and bereavement-related information. Pearson’s correlation coefficient was used to explore the associations between perceived stress, anxiety, social support and PGD. Multiple imputation was used to replace the missing data. Then we employed the SPSS PROCESS macro to test the mediation model and the moderated mediation model [49]. First of all, Model 4 was used to determine the mediation role of anxiety in the relation between perceived stress and PGD. Next, Model 59 was adopted to explore whether social support moderated the direct and the indirect effects of perceived stress on PGD. Finally, the significance of the moderation effect was examined by a simple-slope test which used the PROCESS macro. We kept chronic disease, annual household income, debt, religious belief, marital satisfaction and cause of death as potential confounders in the whole analysis, and categorical variables were converted into dummy variables. All the predictors were standardized to avoid multi-collinearity effects. In the study, on the basis of 5000 bootstrap resamples of these data, 95% bias-corrected confidence intervals (CIs) of the effect were computed. If 95% CIs don’t contain 0 that indicate significant effects. A two-sided *p* < 0.05 was regarded as statistically significant.

## Results

### Preliminary analysis

Since the present study collected data through self-reporting measurements, we employed Harman’s single-factor test to examine common method biases [50]. The results showed that eight factors’ eigenvalues were greater than one and the first factor could explain 34.30% of the variability in the data, which was less than the critical threshold of 40%, indicating that there was no serious common method bias.

### Participants’ characteristics

Table 1 shows the demographic characteristics and bereavement-related information and results of univariate analysis. The mean age of the subjects was 61.06 ± 6.72 years (ranging from 25 to 84). The sample comprised 204 (40.40%) males and 301 (59.60%) females. The mean total score of PG-13 was 26.26 ± 9.67 (ranging from 11-55). Of the 505 participants, 9.11% (*n* = 46) met the criteria for PGD symptoms. In this study, results from the univariate analysis presented that chronic disease, annual household income, debt, religious belief, marital satisfaction and cause of death were significantly associated with PGD symptoms (*p* < 0.05), and these variables were kept as potential confounders in the mediation and the moderated mediation analyses.

**Table 1:** Demographic characteristics and results of univariate analysis

**Variables**	***N (%)***	**PGD symptoms mean (SD)**	***t/F***	***p***
**Gender**			1.08	0.30
Male	204 (40.40)	25.72 (9.44)		
Female	301 (59.60)	26.62 (9.82)		
**Age**			1.91	0.15
<60	193 (38.22)	26.07 (9.38)		
60-69	258 (51.09)	26.79 (10.04)		
≥70	41 (8.12)	23.68 (8.21)		
Missing	13 (2.57)			
**Marital status**			0.53	0.67
Married	318 (62.97)	26.54 (9.30)		
Divorced	134 (26.53)	25.38 (9.90)		
Widowed	49 (9.70)	26.88 (11.30)		
Other	4 (0.79)	25.75 (11.47)		
**Education level**			1.63	0.20
Middle school or under	336 (66.53)	25.89 (9.66)		
Senior high school	140 (27.72)	26.36 (8.97)		
Undergraduate or above	29 (5.74)	30.07 (12.26)		
**Employment**			2.00	0.14
Unemployment	407 (80.59)	26.63 (9.81)		
Part-time	58 (11.49)	24.98 (8.41)		
Full-time	28 (5.54)	23.46 (9.65)		
Missing	12 (2.38)			
**Chronic disease**			11.80	<0.01
No	226 (44.75)	24.63 (8.95)		
Yes	279 (55.25)	27.57 (10.04)		
**Annual household income**			17.92	<0.01
≤10000	42 (8.32)	35.36 (9.00)		
10000-30000	230 (45.54)	26.53 (9.65)		
30000-50000	190 (37.62)	23.88 (8.62)		
≥50000	43 (8.51)	26.37 (9.55)		
**Debt**			22.56	<0.01
No	466 (92.28)	25.57 (8.96)		
Yes	29 (5.74)	37.55 (13.40)		
Missing	10 (1.98)			
**Religious belief**			4.90	0.03
No	476 (94.26)	26.06 (9.57)		
Yes	21 (4.16)	30.81 (10.95)		
Missing	8 (1.58)			
**Marital satisfaction**			4.63	0.01
Unsatisfactory	84 (16.63)	28.90 (10.26)		
General	187 (37.03)	25.14 (9.57)		
Satisfactory	220 (43.57)	26.17 (9.35)		
Missing	14 (2.77)			
**Gender of the deceased child**			1.89	0.17
Male	327 (64.75)	25.83 (9.67)		
Female	172 (34.06)	27.08 (9.64)		
Missing	6 (1.19)			
**Age of the deceased child**			2.53	0.06
<5	29 (5.74)	23.07 (9.06)		
5-15	46 (9.11)	27.32 (10.66)		
16-25	196 (38.81)	25.41 (8.17)		
≥26	227 (44.95)	27.19 (10.60)		
Missing	7 (1.39)			
**Time since the child’s death**			2.54	0.06
≤5	201 (39.80)	27.55 (10.13)		
6-10	87 (17.23)	25.99 (8.57)		
11-15	92 (18.22)	25.84 (10.24)		
≥16	117 (23.17)	24.56 (8.95)		
Missing	8 (1.58)			
**Cause of death**			4.15	<0.01
Accident	132 (26.14)	27.60 (9.31)		
Acute disease	197 (39.01)	27.12 (9.80)		
Chronic disease	150 (29.70)	24.39 (9.59)		
Mental disease	21 (4.16)	23.00 (9.14)		
Missing	5 (0.99)			
**Whether the participant had a grandchild**			0.34	0.56
No	425 (84.16)	26.17 (9.63)		
Yes	57 (11.29)	26.96 (10.00)		
Missing	23 (4.55)			

### Correlations among all the study variables

Correlations among PGD, perceived stress, anxiety and social support are presented in Table 2. Perceived stress and anxiety were positively related to PGD symptoms (*r* = 0.62, *p* < 0.01; *r* = 0.64, *p* < 0.01). Social support was negatively associated with perceived stress, anxiety and PGD symptom (*r* = -0.47, *p* < 0.01; *r* = -0.43, *p* < 0.01; *r* = -0.44, *p* < 0.01). In addition, perceived stress had a significant positive relation to anxiety (*r* = 0.62, *p* < 0.01).

**Table 2:** Correlations among PGD, perceived stress, anxiety and social support

Variables	1	2	3	4
Perceived stress	1			
Anxiety	0.62**	1		
Social support	-0.47**	-0.43**	1	
PGD	0.62**	0.64**	-0.44**	1
M	19.82	51.19	23.70	26.26
SD	5.38	11.98	8.07	9.67

^*^^*^ *p* < 0.01 (two tailed)

### Mediation analysis

As shown in Table 3, after controlling for chronic disease, annual household income, debt, religious belief, marital satisfactory and cause of death, the direct and the total effects of perceived stress on PGD were significant (*β* = 0.62, *p* < 0.01; *β* = 1.02, *p* < 0.01), which proved perceived stress significantly and positively correlated with PGD. The indirect effect of perceived stress on PGD via anxiety was also significant (*β* = 0.40, *p* < 0.01), and the Bootstrap 95% CI for the indirect effect of anxiety was [0.29, 0.52] that didn’t include 0. The mediation effect accounted for 39.22% of the total effect of the relationship between perceived stress and PGD. Thus, anxiety was proved to partially mediate the relationship between perceived stress and PGD symptoms.

**Table 3:** Mediation analysis

**Dependent Variables**	PGD				Anxiety				PGD			
	*β*	*SE*	*t*	*p*	*β*	*SE*	*t*	*p*	*β*	*SE*	*t*	*p*
**Chronic disease**	-0.52	0.69	-0.76	0.45	2.64**	0.85	3.09	<0.01	-1.38*	0.63	-2.17	0.03
**Annual household income**												
10000-30000 vs. <10000	-1.90	1.33	-1.43	0.15	-1.55	1.65	-0.94	0.35	-1.39	1.22	-1.15	0.25
30000-50000 vs. <10000	-3.08*	1.37	-2.25	0.02	-3.05	1.71	-1.79	0.07	-2.09	1.26	-1.66	0.10
>50000 vs. <10000	-2.71	1.69	-1.60	0.11	-4.38*	2.10	-2.08	0.04	-1.29	1.56	-0.83	0.41
**Debt**	6.04**	1.50	4.02	<0.01	3.40	1.87	1.82	0.07	4.93**	1.38	3.58	<0.01
**Religious belief**	3.82*	1.66	2.31	0.02	5.74**	2.06	2.78	<0.01	1.96	1.53	1.28	0.20
**Marital satisfaction**												
General vs. unsatisfactory	-0.27	0.98	-0.28	0.78	0.79	1.22	0.65	0.52	-0.53	0.90	-0.59	0.56
Satisfactory vs. unsatisfactory	1.25	0.97	1.28	0.20	-0.71	1.21	-0.58	0.56	1.48	0.89	1.65	0.10
**Cause of death**												
Acute disease vs. accident	1.16	0.84	1.38	0.17	2.76**	1.05	2.64	<0.01	0.26	0.78	0.34	0.74
Chronic disease vs. accident	-1.68	0.89	-1.89	0.06	1.15	1.10	1.04	0.30	-2.05*	0.81	-2.52	0.01
Mental disease vs. accident	-0.78	1.74	-0.45	0.65	-0.38	2.17	-0.17	0.86	-0.66	1.60	-0.41	0.68
**Perceived stress**	1.02**	0.07	15.03	<0.01	1.23**	0.08	14.60	<0.01	0.62**	0.07	8.33	<0.01
**Anxiety**									0.32**	0.03	9.79	<0.01
**R**	0.66				0.66				0.73			
**R**^**2**^	0.44				0.43				0.53			
**F**	31.86				31.30				42.46			

^*^ *p* < 0.05, ** *p* < 0.01 (two tailed)

### Moderated mediation analysis

**Fig. 2:**
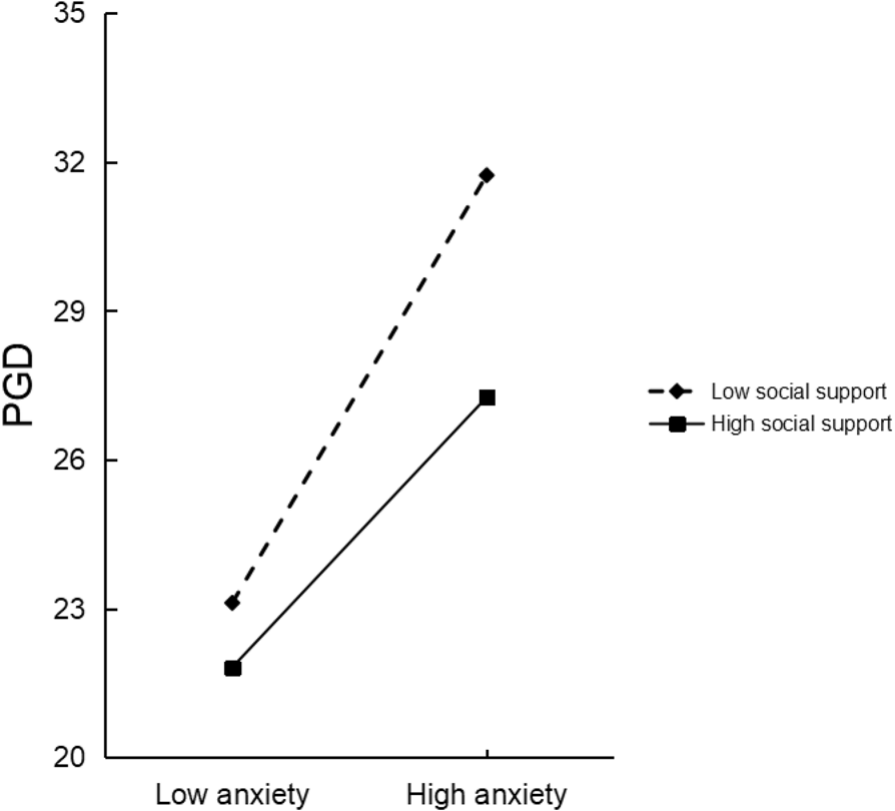
Plot of the relationship between anxiety and PGD at two levels of social support

The PROCESS macro (model 59) by Hayes [49] was used to test the moderated mediation model. After controlling for chronic disease, annual household income, debt, religious belief, marital satisfactory and cause of death, Table 4 shows the interaction effect of anxiety and social support was negatively correlated with PGD (*β* = -0.01, *p* = 0.04), indicating social support moderated the pathway of “anxiety-PGD”. Nevertheless, the interaction effect of perceived stress and social support was insignificantly correlated with anxiety (*β* = 0.01, *p* = 0.15) and PGD (*β* < 0.01, *p* = 0.72), indicating social support did not moderate the pathway of “perceived stress-anxiety” and the direct pathway of “perceived stress-PGD”. To better explain the moderating effect of social support, we divided social support into three different levels (low, 1 SD below mean; moderate, mean; high, 1 SD above mean). Anxiety significantly mediated the link between perceived stress and PGD when social support was low (*β* = 0.36, 95%CI: 0.24, 0.49), moderate (*β* = 0.32, 95%CI: 0.22, 0.45) and high (*β* = 0.27, 95%CI: 0.13, 0.47). Figure 2 illustrates the plot of the link between anxiety and PGD at two levels of social support (1 SD below mean, 1SD above mean). The results indicated that anxiety was strongly associated with PGD for participants with low social support (*β* = 0.36, SE = 0.04, *t* = 8.52, *p* < 0.01), while the association was weaker for participants with high social support (*β* = 0.23, SE = 0.05, *t* = 4.57, *p* < 0.01).

**Table 4:** Moderated mediation analysis

**Dependent Variables**	Anxiety				PGD			
	*β*	*SE*	*t*	*p*	*β*	*SE*	*t*	*p*
**Chronic disease**	2.34**	0.84	2.78	<0.01	-1.42*	0.62	-2.28	0.02
**Annual household income**								
10000-30000 vs. <10000	-1.38	1.65	-0.84	0.40	-0.74	1.21	-0.61	0.54
30000-50000 vs. <10000	-3.02	1.71	-1.77	0.08	-1.45	1.26	-1.15	0.25
>50000 vs. <10000	-3.62	2.11	-1.72	0.09	-0.27	1.55	-0.17	0.86
**Debt**	3.35	1.84	1.82	0.07	4.72**	1.35	3.49	<0.01
**Religious belief**	5.84**	2.03	2.87	<0.01	2.70	1.51	1.79	0.07
**Marital satisfaction**								
General vs. unsatisfactory	1.08	1.20	0.90	0.37	-0.33	0.89	-0.37	0.71
Satisfactory vs. unsatisfactory	-0.17	1.20	-0.14	0.89	1.88*	0.88	2.13	0.03
**Cause of death**								
Acute disease vs. accident	2.76**	1.03	2.67	<0.01	0.40	0.76	0.52	0.60
Chronic disease vs. accident	1.17	1.10	1.08	0.28	-1.82*	0.80	-2.28	0.02
Mental disease vs. accident	0.01	2.14	<0.01	1.00	-0.79	1.57	-0.51	0.61
**Perceived stress**	1.10**	0.09	12.15	<0.01	0.55**	0.08	7.13	<0.01
**Anxiety**					0.29**	0.03	8.73	<0.01
**Social support**	-0.23**	0.06	-4.06	<0.01	-0.18**	0.04	-4.13	<0.01
**Perceived stress ×social support**	0.01	0.01	1.45	0.15	<0.01	0.01	0.35	0.72
**Anxiety × social support**					-0.01*	<0.01	-2.10	0.04
**R**	0.67				0.74			
**R**^**2**^	0.45				0.55			
**F**	29.05				37.20			

^*^ *p* < 0.05, ** *p* < 0.01(two tailed)

## Discussion

The current study examined that anxiety partially mediated the link between perceived stress and PGD as well as social support moderated the second stage of indirect effect, which did not moderate the direct effect and the first stage of indirect effect. Furthermore, the relationship between anxiety and PGD was stronger for Shidu parents with lower levels of social support than for those with higher levels of social support. The current study is the first investigation used moderated mediation analysis to reveal the mediation effect of anxiety and the moderation effect of social support in the association between perceived stress and PGD symptoms among Chinese Shidu parents, and the findings promoted a more profound understanding of how and when perceived stress is connected with PGD.

We found perceived stress was positively and directly correlated with PGD symptoms. The finding is consistent with a prior research documenting that when the families who undergo acute bereavement felt more stress about this bereavement, they would tend to suffer stronger grief reactions [25]. Our finding is also in line with the stress and coping theory [27]. After the loss of the only child, Shidu parents should deal with many daily troubles generated in living with the loss. First of all, the core of Chinese traditional culture is Confucianism, which advocates filial piety. Affected by this thought, people believe that a person must have descendants to inherit the family blood, and Shidu parents will be stigmatized by the public as “those who are about to become extinct” and suffer social discrimination [5]. Additionally, the key to maintaining a stable sense of self and self-esteem for Chinese people lies in having close relationships with family members [4]. Hence, it is understandable that the death of an only child will make Shidu parents feel low esteem so that engaging in less social interaction. Finally, there is a Chinese saying, “bringing up children for one's old age”, which means children are primary caregivers for the elderly in China. Losing the only child will leave parents facing the plight of old age without support. After retirement, the elderly who lost their only child with low incomes may only maintain a basic life [51]. With the growth of age, they will suffer from more diseases and bear great financial pressure from the increasing cost of medical and care expenses [51]. When the traumatic loss and the daily troubles are appraised by Shidu parents as taxing or exceeding their available resources, they can feel stressed. During the subsequent coping process, Shidu parents may lose their sense of control over life due to their inability to resolve these stressful events, withdraw from the outside world, and continue to immerse in the death of the child.

As expected, our study showed anxiety partially mediated the relation between perceived stress and PGD in Shidu parents. To be specific, a higher level of perceived stress may cause higher levels of anxiety and further bring about higher levels of PGD symptoms. For the correlation between perceived stress and anxiety, our result is in line with previous research that showed a positive association between them [33, 34, 52]. The potential mechanism for this may be that stress directly related to the traumatic loss and realistic stressors such as financial adversity and social isolation can trigger anxiety associated with a multitude of possible challenging new demands [53]. The former type of stress can leave the bereaved with anxiety symptoms such as feelings of insecurity, helplessness and the unpredictability of life; and the latter type of stress can exacerbate these feelings [53]. Meanwhile, with perceived stress increasing, individuals will engage in less exercise or more sedentary behavior, leading to negative outcomes [54]. For the association between anxiety and PGD symptoms, our study coincides with prior research suggesting that anxiety displayed significant positive correlation with PGD symptoms [18, 35]. This may be due to when anxiety is severe and intense, continued troubling concerns about the consequences of bereavement, dysregulated emotions and excessive avoidance behaviors can affect the bereaved's acceptance of the loss and inability to find meaning in life without the deceased, which will disrupt the natural healing process of grief, then grief reaction may become complicated and prolonged [53]. On the basis of the above prior research indicating the correlation between perceived stress and anxiety as well as the correlation between anxiety and PGD symptoms, the current study further reveals Shidu parents who feel more stress have more anxiety and are more likely to suffer from PGD.

Moreover, the results showed that social support moderated the second stage of the indirect effect. Compared with participants reporting higher levels of social support, the association between anxiety and PGD was closer for those with lower levels of social support. The finding is congruent with prior research, which found that when nurses perceived lower social support, the higher death anxiety was connected with higher psychological distress [55]. This finding also supports the biobehavioral attachment-based model of prolonged grief that a good social support system helps Shidu parents stop avoiding the loss of their only child and adapt to life without the child [32]. There are two possible explanations for the moderate role of social support on the relationship between anxiety and PGD symptoms. Firstly, significant others’ caring, comforting and material assistance to Shidu parents can help directly reduce their situational needs, diminish physical arousal and unease as well as support the belief that they do belong to and are accepted with a caring network [36, 56]. Secondly, social support also promotes individuals to confirm the sense of true importance to others and maintain their sense of self-worth [37]. However, we found that social support did not moderate the first half of indirect effect of perceived stress on PGD, which was not consistent with some previous findings [38, 39]. One possible explanation is the difference of research subjects (parturient, frontline COVID-19 medical staff vs. Shidu parent). Losing the only child is one of the most stressful experiences, dealing with anxiety resulted from this traumatic stressor, high social support may be not enough. Moreover, because the current study was a cross-sectional study, we only measured Shidu parents’ perceived level of social support after the loss, social support data before the death of their children is lacking, maybe long-term continuous measurement of social support can make the results more accurate.

Several limitations of the current study should be outlined. First of all, this study was based on a cross-sectional design, which cannot be employed to determine the causal relationships among perceived stress, anxiety, social support and PGD symptoms. Future studies could adopt a longitudinal design to address the limitation. Second, the assessments were founded on self-reporting, instead of the standard clinical interview, and response bias was unavoidable. Third, the sample in the current research were recruited from urban districts in Shenyang, China, which might have difficulty in representing people from other areas or diverse cultural backgrounds in China. Future studies should expand the sample size to better represent the huge population of Shidu parents in China.

There are some implications in the study. First, losing the only child could make parents suffer from various stresses, including psychological, economic and social stresses [57]. Previous studies have explored high levels of perceived stress may not only relate to emotional disturbances such as anxiety, depression, PTSD and PGD symptoms [57–60], but also result in physical illnesses such as cardiovascular disease [61]. Additionally, results of a study by Alkhawaldeh et al. [62] showed the stress management program (including psychoeducation lecture, relaxation technique training, cognitive restructuring, etc.) was an effective method that could be employed to reduce stress levels among public health nurses. Thus, early screening and appropriate interventions for individuals with higher stress levels to improve their mental health is essential. Second, since social support could buffer the indirect link between perceived stress and PGD symptoms via anxiety, improving social support would protect individuals from suffering from PGD. Therefore, it is necessary for the government to provide Shidu parents with objective support, such as financial aid, pension services and medical security. In addition, it is also vital for the community to provide more specialized mental health services from counsellors, social workers, psychologists and psychiatrists to Shidu parents [15].

## Conclusions

The study concluded anxiety partly mediated the association between perceived stress and PGD, and social support moderated the second half of the indirect effect of the mediating model. Furthermore, the results highlighted the significance of decreasing perceived stress and enhancing social support in preventing PGD symptoms among Shidu parents.

## Data Availability

The datasets used and analyzed during the current study are available from the corresponding author (Yang Wang) on reasonable request.

## Abbreviations

PGD: Prolonged grief disorder
